# Porcine Plasmacytoid Dendritic Cells Are Unique in Their Expression of a Functional NKp46 Receptor

**DOI:** 10.3389/fimmu.2022.822258

**Published:** 2022-03-11

**Authors:** Kerstin H. Mair, Maria Stadler, Mahsa Adib Razavi, Armin Saalmüller, Wilhelm Gerner

**Affiliations:** ^1^ Institute of Immunology, Department of Pathobiology, University of Veterinary Medicine Vienna, Vienna, Austria; ^2^ Christian Doppler (CD) Laboratory for Optimized Prediction of Vaccination Success in Pigs, Institute of Immunology, Department of Pathobiology, University of Veterinary Medicine Vienna, Vienna, Austria

**Keywords:** NKp46, plasmacytoid dendritic cells, swine, interferon-α, pS6

## Abstract

The activating receptor NKp46 shows a unique expression pattern on porcine leukocytes. We showed already that in swine not all NK cells express NKp46 and that CD3^+^NKp46^+^ lymphocytes form a T-cell subset with unique functional properties. Here we demonstrate the expression of NKp46 on CD4^high^CD14^-^CD172a^+^ porcine plasmacytoid dendritic cells (pDCs). Multicolor flow cytometry analyses revealed that the vast majority of porcine pDCs (94.2% ± 4) express NKp46 *ex vivo* and have an increased expression on the single-cell level compared to NK cells. FSC/SSC^high^CD4^high^NKp46^+^ cells produced high levels of IFN-α after CpG ODN 2216 stimulation, a hallmark of pDC function. Following receptor triggering with plate-bound monoclonal antibodies against NKp46, phosphorylation of signaling molecules downstream of NKp46 was analyzed in pDCs and NK cells. Comparable to NK cells, NKp46 triggering led to an upregulation of the phosphorylated ribosomal protein S6 (pS6) in pDCs, indicating an active signaling pathway of NKp46 in porcine pDCs. Nevertheless, a defined effector function of the NK-associated receptor on porcine pDCs could not be demonstrated yet. NKp46-mediated cytotoxicity, as shown for NK cells, does not seem to occur, as NKp46^+^ pDCs did not express perforin. Yet, NKp46 triggering seems to contribute to cytokine production in porcine pDCs, as induction of TNF-α was observed in a small pDC subset after NKp46 cross-linking. To our knowledge, this is the first report on NKp46 expression on pDCs in a mammalian species, showing that this receptor contributes to pDC activation and function.

## Introduction

Dendritic cells (DCs) are part of the innate immune system and play a central role in antigen presentation to T cells. Plasmacytoid dendritic cells (pDCs) are a small and highly specialized cell subset belonging to the DC family (1). They express high levels of the intracellular toll-like receptors (TLR) 7 and 9, which recognize single-stranded RNA or unmethylated CpG motif-containing DNA, leading to induction of interferon (IFN)-α production by pDCs (2). As they are the major source of IFN-α, they were also designated as natural interferon-producing cells.

In the pig, pDCs likewise represent a small subset with frequencies below 1% in peripheral blood mononuclear cells (PBMC), lymph nodes, and tonsils. Nevertheless, porcine pDCs seem to be more abundant (5%–6%) in the spleen (3, 4). Phenotypically, porcine pDCs are identified as CD3^-^CD4^high^CD14^-^CD16^+^CD172a^low^ myeloid cells (3, 5) and express low to moderate levels of MHC-II (6, 7). Additionally, porcine pDCs express CD135 (Flt3), a tyrosine kinase that is crucial for pDC development by binding its ligand (Flt3L) as shown for human and mouse (8). Transcriptome analysis of porcine pDCs showed that the gene expression profile was very similar to their human and murine counterparts (7, 9). Porcine pDCs are able to produce high amounts of IFN-α in response to *in vitro* stimulation with TLR agonists like imiquimod and CpG oligodeoxynucleotides (ODN) (7, 10). Induction of IFN-α was also observed after *in vitro* stimulation with viruses like the transmissible gastroenteritis coronavirus (3, 10) or *ex vivo* in pDC and sera of pigs experimentally infected with the classical swine fever virus (11). Stimulation and increased production of IFN-α by pDCs were detected in pigs after foot-and-mouth disease virus (FMDV) infection *in vitro* when FMDV was complexed with virus-specific immunoglobulins (12, 13). In contrast, wild-type pseudorabies virus infection leads to a suppression in IFN-α production by porcine pDCs after infection compared to using an attenuated vaccine strain (14). Although suppression of pDCs by the porcine reproductive and respiratory syndrome virus (PRRSV) was shown (10), more recent studies showed that PRRSV inhibition of IFN-α production from pDCs was weak or absent and dependent on the genotype of PRRSV (15, 16). Furthermore, it could be shown that pDC stimulation was stronger by using PRRSV-infected cells than direct stimulation by virions (16). Hence, as shown in human and mouse, porcine pDCs appear to be major IFN-α producers following viral infection.

The activating receptor NKp46 (NCR1, CD335) is used as a marker for the identification of natural killer (NK) cells in various mammalian species (17). NKp46 is a type I transmembrane glycoprotein, and signaling is mediated by the adaptor proteins CD3ζ and FcϵRIγ (18, 19). Receptor triggering leads to Ca^2+^ induction driving cytotoxicity and cytokine production (20). Known ligands for NKp46 are hemagglutinins of influenza, parainfluenza, or Sendai virus (21, 22) as well as the natural ligand vimentin that is upregulated on *Mycobacterium tuberculosis*-infected cells (23). NKp46 shows a unique expression pattern on porcine lymphocytes. NKp46 expression on porcine NK cells separates this lymphocyte population in three distinct subsets: NKp46^-^, NKp46^+^, and NKp46^high^ CD3^-^ lymphocytes that have phenotypic and functional properties of NK cells (24–26). Furthermore, a population of CD3^+^NKp46^+^ cells was also identified in the pig, which has phenotypical properties of T cells but functionally resembles NK cells (27). Triggering of NKp46 on porcine NK cells leads to cytotoxicity and IFN-γ production (25, 26). Likewise, NKp46 triggering leads to degranulation of CD3^+^NKp46^+^ cells (27).

Up to date, there is no report on NKp46 expression on porcine myeloid cells. Here, we identify NKp46 expression on a subset of porcine myeloid cells that have the phenotype of pDCs and produce IFN-α after *in vitro* stimulation. Our data show that the vast majority of porcine pDCs express this “NK-cell associated” receptor at high levels and triggering of NKp46 leads to the induction of downstream signaling events, indicating a functional role of this receptor on porcine pDCs. Thus, porcine NKp46 seems to have a unique expression pattern in porcine leukocytes compared to other species and our data suggest an additional role for this receptor in innate immune sensing in the pig.

## Material and Methods

### Isolation and Cell Culture of Porcine PBMC

Blood was obtained from healthy 3–7-month-old pigs from an abattoir in Austria. Animals were subjected to electric high-voltage anesthesia followed by exsanguination, a procedure that is in accordance with the Austrian Animal Welfare Slaughter Regulation. Blood from 5-week-old piglets was obtained from animals housed at the University Clinic for Swine at the University of Veterinary Medicine Vienna. Animals were anaesthetized by intramuscular injection of Ketaminhydrochlorid (Narketan^®^, Vétoquinol, Vienna, Austria, 10 mg/kg body weight) and Azaperone (Stresnil^®^, Janssen Pharmaceutica, Beerse, Belgium, 1.3 mg/kg body weight). Subsequently, animals were euthanized *via* intracardial injection of T61^®^ (MSD Animal Health, Vienna, Austria, 1.0 ml/10 kg body weight). This procedure was approved by the institutional ethics committee and the national authority according to § 26 of Law for Animal experiments, Tierversuchsgesetz 2012 – TVG 2012 (reference number: bmwf GZ68.205/0005-II/3b/2014). All animals used for sample collection were clinically healthy, and no pathological indications were observed at necropsy. PBMC were isolated from heparinized blood using density gradient centrifugation (Pancoll human, density: 1.077 g/ml, PAN-Biotech, Aidenbach, Germany), and isolated cells were stored at -150°C. For *ex vivo* analyses, PBMC were used immediately after thawing. When cryopreserved cells were subjected to *in vitro* stimulation, PBMC were thawed 1 day prior to stimulation and rested overnight in RPMI 1640 with stable glutamine (PAN-Biotech) supplemented with 10% (v/v) heat-inactivated FCS (PAN-Biotech), 100 IU/ml penicillin, and 0.1 mg/ml streptomycin (PAN-Biotech).

### Stimulation of pDCs and NK Cells for Detection of Intracellular Signaling Molecules and Cytokines

For virus stimulation, cells were either cultured in the presence of the influenza isolate A/swine/Germany/AR1190/2014 (H1N2) at a MOI of 0.5 (GISAID EpiFlu database accession number EPI_ISL_222085) or cultivated in the presence of a corresponding mock control. For TLR stimulation, cells were stimulated with 5 µg/ml of a TLR9-agonist class A oligonucleotide (ODN) or corresponding control (**Table 1**). For all stimulations, cells were cultured in 96-well round-bottom plates with a final cell number of 3 × 10^5^ cells in 200 µl per well. Receptor triggering was performed by using mAbs against NKp46 (mouse IgG1, clone VIV-KM3, in-house production). Isotype-matched irrelevant antibodies (clone NCG01, Dianova, Hamburg, Germany) served as control. 96-well round-bottom plates were coated with mAbs at a concentration of 3 µg/ml in PBS (50 µl per well) overnight at 4°C. Plates were washed three times with PBS prior to addition of 3 × 10^5^ cells in a total volume of 200 µl per well. Cells used for NK-cell receptor-triggering assays were additionally pre-activated with a combination of rpIL-2 (10 ng/ml, R&D Systems, Minneapolis, MN, USA) and rpIL-15 (10 ng/ml, Kingfisher Biotech, Saint Paul, MN, USA) for 24 h. The expression of phosphorylated ribosomal protein S6 (pS6) was analyzed after 3 and 6 h. For intracellular cytokine staining, Brefeldin A (GolgiPlug, BD Biosciences, San Jose, CA, USA) was added to microcultures at a final concentration of 1 µg/ml after 2 h of stimulation for an additional 4 h. Antibodies and reagents used for flow cytometry (FCM) staining are outlined in **Table 2**.

**Table 1:** Synthetic oligonucleotides (ODNs) used for *in vitro* stimulation.

ODN	Sequence (5′–3′)	Source
2216	ggG GGA CGA TCG TCg ggg gg	IBA GmbH, Göttingen, Germany
		InvivoGen, Toulouse, France
2243 (negative control)	ggG GGA GCA TGC TGg ggg gg	IBA GmbH, Göttingen, Germany
		InvivoGen, Toulouse, France

Bases shown in capital letters indicate phosphodiester linkage 3′ of the base; those in lowercase letters indicate phosphorothioate linkages.

**Table 2:** Primary antibodies and secondary reagents used for FCM analyses.

Antigen	Clone	Isotype	Fluorochrome	Labeling strategy	Source of primary Ab
*Ex vivo* phenotyping					
NK cells					
CD3	BB23-8E6	IgG2b	Alexa647	Secondary antibody^a)^	Southern Biotech
CD8α	11/295/33	IgG2a	BV421	Biotin^b)^-streptavidin^c)^	In-house
CD16*	G7	IgG1	PE	Secondary antibody^d)^	Bio-Rad^e)^
NKp46*	VIV-KM1	IgG1	PE	Secondary antibody^d)^	In-house
NKp44*	54-1	IgG1	PE	Secondary antibody^d)^	In-house^f)^
perforin*	Pf344	IgG1	PE	Secondary antibody^d)^	Mabtech^g)^
					
pDC panel I					
CD4	74-12-4	IgG2b	FITC	Directly conjugated	BD Biosciences
CD172a	74-22-15A	IgG2b	BV421	Biotin^b)^-Streptavidin^c)^	In-house
CD16*	G7	IgG1	PE	Secondary antibody^d)^	Bio-Rad
NKp46*	VIV-KM1	IgG1	PE	Secondary antibody^d)^	In-house
NKp44*	54-1	IgG1	PE	Secondary antibody^d)^	In-house^f)^
perforin*	Pf344	IgG1b	PE	Secondary antibody^d)^	Mabtech
					
pDC panel II					
CD4	74-12-4	IgG2b	FITC	Directly conjugated	BD Biosciences
NKp46	VIV-KM1	IgG1	Alexa647	Directly conjugated^h)^	In-house
CD14*	CAM36A	IgG1	PE	Secondary antibody^d)^	Kingfisher Biotech
CD16*	G7	IgG1	PE	Secondary antibody^d)^	Bio-Rad
CD163*	2A10/11	IgG1	PE	Directly conjugated	Bio-Rad
CD172a*	74-22-15	IgG1	PE	Secondary antibody^d)^	In-house
					
Intracellular cytokine staining					
pDCs					
CD4	74-12-4	IgG2b	Alexa488	Secondary antibody^i)^	In-house
CD172a	74-22-15	IgG1	eFluor450	Biotin^b)^-streptavidin^j)^	In-house
NKp46	VIV-KM1	IgG1	PE	Secondary antibody^d)^	In-house
IFN-α*	F17	IgG1	Alexa647	Directly conjugated^h)^	Thermo Fisher Scientific
IFN-γ*	CC302	IgG1	Alexa647	Directly conjugated	Bio-Rad
TNF-α	MAb11	IgG1	BV605	Directly conjugated	BioLegend
Phospho-specific staining unsorted cells					
NK cells					
CD3	BB23-8E6	IgG2b	Alexa647	Secondary antibody^a)^	Southern Biotech
CD8α	11/295/33	IgG2a	eFluor450	Biotin^b)^-Streptavidin^k)^	In-house
pS6_(S235/S236)_	cupk43k	IgG1	PE	Directly conjugated	Thermo Fisher Scientific
					
pDCs					
CD4	74-12-4	IgG2b	Alexa488	Secondary antibody^k)^	In-house
CD172a	74-22-15	IgG1	Alexa647	Directly conjugated^h)^	In-house
pS6_(S235/S236)_	cupk43k	IgG1	PE	Directly conjugated	Thermo Fisher Scientific
					
FACS Sort					
CD4	74-12-4	IgG2b	PerCP-Cy5.5	Directly conjugated	BD Biosciences
CD8α	11/295/33	IgG2a	BV421	Secondary antibody^k)^	In-house
CD172a	74-22-15A	IgG2b	Alexa647	Biotin^b)^-Streptavidin^l)^	In-house

^a^Goat anti-mouse anti-IgG2b-Alexa647, Thermo Fisher Scientific.

^b^EZ-Link™ Sulfo-NHS-LC-Biotin, Thermo Fisher Scientific.

^c^Streptavidin-Brilliant Violet 421, BioLegend, San Jose, CA, USA.

^d^Goat anti-mouse anti-IgG1-PE, Southern Biotech, Birmingham, AL, USA.

^e^Bio-Rad, Hercules, CA, USA.

^f^Kindly provided by Joan K. Lunney, Animal Parasitic Disease Laboratory, BARC, ARS, USDA, Beltsville, MD, USA (28).

^g^Mabtech, Nacka Strand, Sweden.

^h^Alexa Fluor™ 647 Antibody Labeling Kit, Thermo Fisher Scientific.

^i^Goat anti-mouse anti-IgG2b-Alexa488, Thermo Fisher Scientific.

^j^Streptavidin-eFluor450, Thermo Fisher Scientific.

^k^Goat anti-mouse anti-IgG2a-Brilliant Violet 421, Jackson ImmunoResearch, Suffolk, UK.

^l^Streptavidin-Alexa647, Thermo Fisher Scientific.

*Used in different samples.

### Flow Cytometry Assays and Antibodies Used in the Study

For FCM analyses, cells were resuspended either in PBS-based buffer containing 10% (v/v) porcine plasma for *ex vivo* analysis or in buffer containing 3% (v/v) FCS for analysis after *in vitro* cultivation. All incubation steps were performed in 96-well round-bottom plates at 4°C for 20 min. The different combinations of primary monoclonal antibodies (mAbs) as well as secondary reagents used for each assay are listed in **Table 2**. Non-commercial antibodies were produced in-house (29). Where indicated, these antibodies were conjugated either to fluorochromes (Alexa Fluor-647 Protein Labeling Kit, Thermo Fisher Scientific, Vienna, Austria) or to Biotin (EZ-Link Sulfo-NHS-LC-Biotin, Thermo Fisher Scientific) according to the manufacturer’s protocols. If unlabeled and directly conjugated antibodies with the same isotype were used in combination, a sequential staining was performed. After labeling with unconjugated primary mAb and isotype-specific dye-conjugated secondary antibodies, free binding sites were blocked by whole mouse IgG molecules (2 µg per sample, Jackson ImmunoResearch, Suffolk, UK). Thereafter, cells were incubated with directly labeled primary mAbs. For exclusion of dead cells, Fixable Viability Dye (VD) eFluor 780 (Thermo Fisher Scientific) was used according to the manufacturer’s protocol with 0.025 µl reactive dye per sample to discriminate dead cells. Unstimulated cells were used as control staining for activated cells. Single-color samples were prepared for automatic compensation.

**Figure 1:**
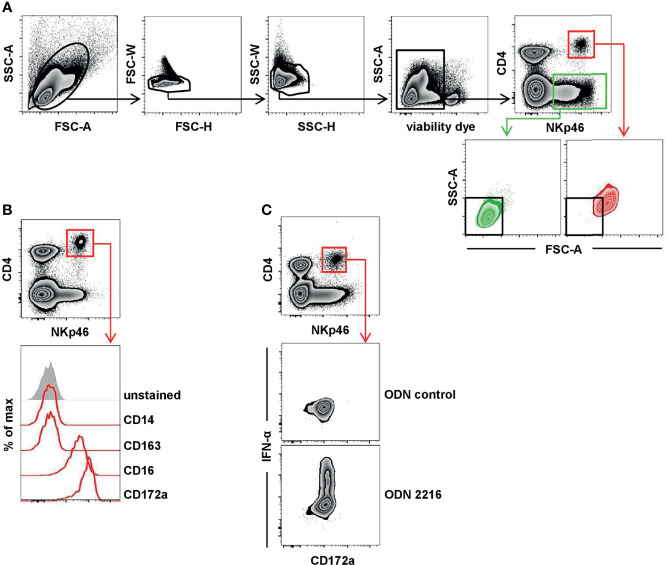
Porcine CD4^high^CD172a^low^ pDC express NKp46. **(A)** Total PBMC were gated according to their light-scatter properties. Potential doublet cells were excluded by consecutive FSC-H/FSC-W and SSC-H/SSC-W gates, followed by gating on cells negative for the viability dye VDeFluor780 to exclude dead cells. Within live PBMC, two NKp46^+^ cell subsets were separated according to their CD4 co-expression: CD4^+^NKp46^+^ (red gate) and CD4^-^NKp46^+^ (green gate). CD4/NKp46-defined subsets were backgated and analyzed for their light-scatter properties. **(B)** CD4^+^NKp46^+^ cells derived from blood (red gate) were analyzed for co-expression of different monocyte/DC-associated surface markers by multicolor FCM. Expression profiles of CD14, CD16, CD163, and CD172a of CD4^+^NKp46^+^ cells from one representative animal are shown as red open histograms. Unstained control is shown as tinted gray histogram on the top. **(C)** PBMC were stimulated either with the TLR9 agonist ODN 2216 or with a control oligonucleotide (ODN control) for 6 h. Following stimulation, intracellular IFN-α expression of CD4^high^CD172a^low^ pDC (red gate) was analyzed. Zebra plots show representative results out of experiments with PBMC from five different animals.

For staining of intracellular signaling molecules, cytokines, or perforin, cells were fixed and permeabilized with the Foxp3 staining buffer set (Thermo Fisher Scientific) after cell-surface staining, followed by an incubation step with either mAbs against pS6 for phospho-specific flow cytometry, mAbs against IFN-α, TNF-α, and IFN-γ for detection of intracellular cytokine production, or mAbs against perforin. FCM analyses were performed on a FACSCanto II (BD Biosciences). At least 5 × 10^5^ cells were recorded per sample. For phospho-specific flow cytometry and intracellular cytokine staining, 1 × 10^6^ cells were recorded. Data were analyzed with FACSDiva software (Version 8.0., BD Biosciences) and FlowJo software (Version 10.2, Tree Star, Ashland, OR). A uniform gating hierarchy was used throughout all experiments (**Figure 1A**) for analyses of total PBMC to exclude potential doublet as well as dead cells.

### Cell sorting

For sorting of pDCs and NK cells, a magnetic-activated cell sort (MACS) followed by a fluorescence-activated cell sort (FACS) of the negative fraction was performed. Freshly isolated PBMC were incubated with mAbs directed against CD3 (clone PPT3, mouse IgG1, in-house) for T-cell depletion. This was followed by incubation with anti-mouse IgG1 magnetic microbeads (Miltenyi Biotec, Bergisch Gladbach, Germany) in a buffer containing PBS (PAN-Biotech) supplemented with 2% FCS (Thermo Fisher Scientific) and 2 mM EDTA. For depletion of CD3^+^ cells, LD columns from Miltenyi Biotec were used according to the manufacturer’s protocol. The negative fraction (purity 96% or higher) was then forwarded to FACS. Cells were incubated with mAbs and secondary reagents, as indicated in **Table 2**. Additionally, cells were stained with the VD as indicated above and goat anti-mouse IgG1-Alexa488 secondary Abs (Thermo Fisher Scientific) to stain residual CD3^+^ cells. Cell sorting was performed on a FACSAria (BD Biosciences). Four different populations were sorted: CD3^-^ (“bulk”), CD3^-^CD8α^-^ (“enriched pDC”), CD3^-^CD4^+^CD172a^+^ (“pDC”), and CD3^-^CD8α^+^ (“NK cells”). The average purity of the sorted cell subsets was 92% or higher. After two washing steps in cell culture medium, sorted cells were cultivated and stimulated for the pS6 assay by plate-bound mAbs, as indicated above. The expression of pS6 was analyzed after 3 h by flow cytometry, as indicated above. Cells of bulk and enriched pDC subset were re-stained with the same panel as used for the FACS sort in addition to the mouse-anti-human/mouse pS6-PE (clone cupk43k, IgG1 isotype, Thermo Fisher Scientific). Pure pDC and NK-cell subsets were stained solely with the pS6-specific mAbs.

### Statistical Analysis

Data were analyzed for statistical significance by SPSS^®^ (SPSS Statistics Version 20.0, IBM Corp., Armonk, NY). Obtained values were tested for normal distribution by the Kolmogorov–Smirnov test. Where required, data sets were subjected to log transformation to meet the condition of normality. Data sets that met the requirement of normal distribution were analyzed by paired two-tailed Student’s t-test; data sets that did not show normal distribution were analyzed by the Wilcoxon signed-rank test. Levels of significance were defined as *p ≤ 0.05* (indicated by *), *p ≤ 0.01* (indicated by **), and *p ≤ 0.001* (indicated by ***). Graphs were prepared using GraphPad Prism V5.04 (GraphPad Software, San Diego, CA, USA).

## Results

### NKp46 Is Expressed on Porcine CD4^+^ Leukocytes With the Phenotype and Function of pDCs

The expression of the activating receptor NKp46 on porcine lymphocytes was described in detail in previous studies (25–27). Besides the expression of NKp46 on classical CD3^-^ NK cells, a CD3^+^NKp46^+^ population of non-conventional T cells has been identified in the pig. Both NKp46^+^ lymphocyte populations showed a CD4^-^ phenotype (25, 27). Interestingly, when analyzing total PBMC, a further distinct population of NKp46^+^ cells could be identified that co-expressed CD4 (**Figure 1A**, red gate). Light-scatter properties in FCM analyses indicated a difference in size between CD4^-^NKp46^+^ (**Figure 1A**, green gate) and CD4^+^NKp46^+^ cells (**Figure 1A**, red gate). CD4^-^ cells could be assigned to the lymphocyte population that include NKp46^+^ NK cells as well as the non-conventional NKp46^+^ T cells. Due to their increased light-scatter properties, we hypothesized that the CD4^+^NKp46^+^ cells refer to dendritic cells or monocytes. Therefore, CD4^+^NKp46^+^ cells were analyzed for co-expression of markers correlated with the myelomonocytic lineage in swine (**Figure 1B**). CD4^+^NKp46^+^ cells showed no expression of the pan-monocyte marker CD14 as well as CD163 that is found on a subset of monocytes in the blood of pigs. In contrast, all CD4^+^NKp46^+^ cells expressed CD16 and CD172a. These findings together with the high CD4 expression point toward the phenotype of porcine pDCs, described as CD4^high^CD14^-^CD16^+^CD172a^low^ cells (5). To corroborate NKp46 expression on this subset further, we performed the FCM staining shown in **Figure 1A** with three different anti-porcine NKp46 mAb clones that were described to recognize different epitopes of the receptor previously (25). All three mAb clones showed the same staining pattern and identified the same frequency of porcine pDCs (**Supplementary Figure 1**).

Porcine pDCs are described as the major IFN-α source in response to TLR9 activation (30, 31). Accordingly, CD4^+^NKp46^+^ cells were analyzed for intracellular cytokine production following stimulation of total PBMC with CpG ODN 2216, which was already shown to be a potent TLR9 agonist for human as well as porcine pDCs. A clear induction of IFN-α was observed within the CD4^+^NKp46^+^ population after ODN 2216 stimulation (**Figure 1C**) in contrast to cells cultivated in the presence of a non-stimulatory ODN control. In parallel, cells were surface stained for CD172a, indicating that all IFN-α^+^ cells also showed the CD172a^dim^ phenotype. These results indicate that CD4^+^NKp46^+^ cells phenotypically as well as functionally resemble porcine pDCs.

### Porcine pDCs and NK Cells Share CD16 But Not NKp44 or Perforin Expression

**Figure 2:**
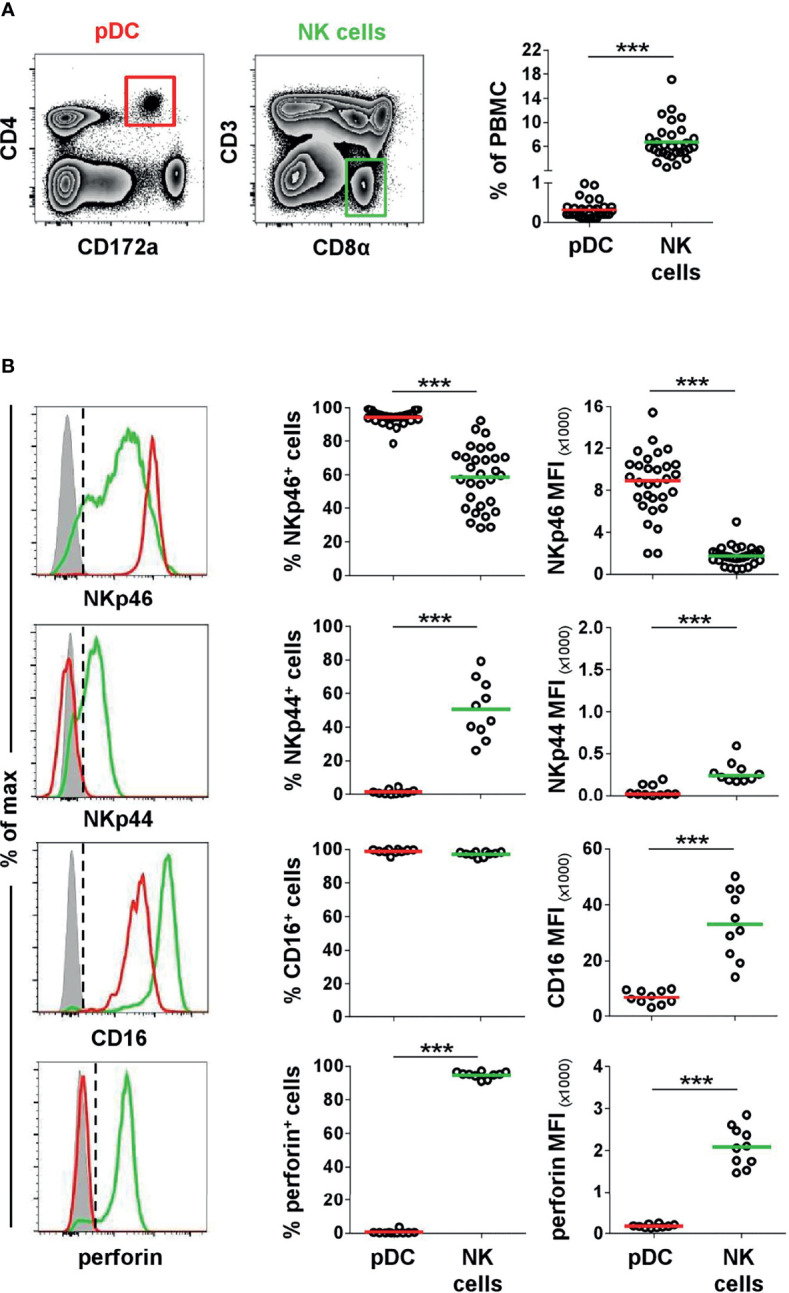
Co-expression of NK-associated markers on porcine pDCs. **(A)** Frequencies of CD4^high^CD172a^low^ pDCs (red gate) and CD3^-^CD8α^+^ NK cells (green gate) were analyzed in blood of 30 animals by FCM. **(B)** Blood-derived porcine pDCs (red) and NK cells (green) were identified by phenotypes as in **(A)** and were analyzed for their expression of the NK-associated surface markers NKp46 (*n = 30*), NKp44 (*n = 10*), and CD16 (*n = 10*) as well as the effector molecule perforin (*n = 10*) by multicolor FCM. Representative staining patterns of one animal are shown in the histograms on the left; unstained control is shown as tinted gray histogram. Percentages of positive cells were obtained according to unstained controls, indicated by the black dashed line. Graphs show frequencies of NKp46^+^, NKp44^+^, CD16^+^, or perforin^+^ cells (middle column), as well as median fluorescence intensities (MFI) of the four molecules (right column) within pDCs and NK cells. Mean (frequencies) and median (MFI) values are represented by colored bars. Significant differences between pDCs and NK cells are indicated (***p ≤ 0.001).

In a next step, we investigated porcine pDCs in more detail in regard to NKp46 expression, as well as the NK-associated molecules NKp44, CD16, and perforin. For this purpose, CD4^high^CD172a^dim^ pDCs (**Figure 2**, red gate and line color) were analyzed by flow cytometry in total PBMC of healthy 3- to 7-month-old pigs. Findings were compared to CD3^-^CD8α^+^ NK cells (**Figure 2**, green gate and line color). As expected, lower frequencies of pDCs were found within PBMC (0.1%–1.0%) compared to NK cells (2.5-17.1%, **Figure 2A**). The expression of NKp46 on porcine NK cells varied between individual animals and frequencies of NKp46^+^ NK cells ranged from 28.4% to 92.2% (**Figure 2B**, upper row). This is consistent with data observed in previous studies showing that also NKp46^-^ NK cells exist in the pig (25). In contrast, the vast majority of porcine CD4^high^CD172a^dim^ cells expressed NKp46 (78.6-99.1%, **Figure 2B**, upper row). Additionally, pDCs also showed on average a 5-fold higher expression level of NKp46 on a per cell level, indicated by the median fluorescence intensity (MFI) of 8,918 ± 2,999, compared to NK cells with a MFI of 1,755 ± 839 (**Figure 2B**, upper row). Additionally, NKp46 expression on pDCs from younger animals (5-week old piglets) was investigated. Overall, reduced frequencies of pDCs within PBMC were observed in animals of this age (**Supplementary Figure 2A, B**, 0.02%–0.3%). Similar to 3–7-month-old pigs, also the majority of piglet-derived pDCs expressed NKp46 (**Supplementary Figure 2B**, 74.6%–98.7%).

Another NCR family member, NKp44, was reported on a minor fraction of pDCs in human (32, 33). Porcine NK cells showed a clear NKp44 expression (26.1%–79.2%), although with a lower MFI (278 ± 130). No obvious NKp44 expression was detected on pDCs (**Figure 2B**, second row). The low-affinity IgG receptor CD16 is another activating receptor expressed by porcine NK cells (25, 26) and was also reported to be expressed on porcine pDCs (5). Comparable high frequencies of CD16^+^ pDCs (95.5%–100%) and NK cells (89.5%–98.8%) could be observed in all animals analyzed (**Figure 2B**, third row). In contrast to NKp46, expression levels for CD16 were 5-fold lower in pDCs (MFI: 6,749 ± 2,530) compared to NK cells (MFI: 32,998 ± 12,350). As those receptors have a function as activating receptors on NK cells and are closely linked to perforin release, we additionally investigated the expression of this effector molecule in both cell populations. As expected, the majority of NK cells expressed perforin (91.0%–97.2%, MFI 2079 ± 472) in contrast to pDCs, where no perforin expression could be detected in all animals analyzed (**Figure 2B**, bottom row).

### Porcine pDCs Produce TNF-α After TLR Stimulation and NKp46 Triggering

**Figure 3:**
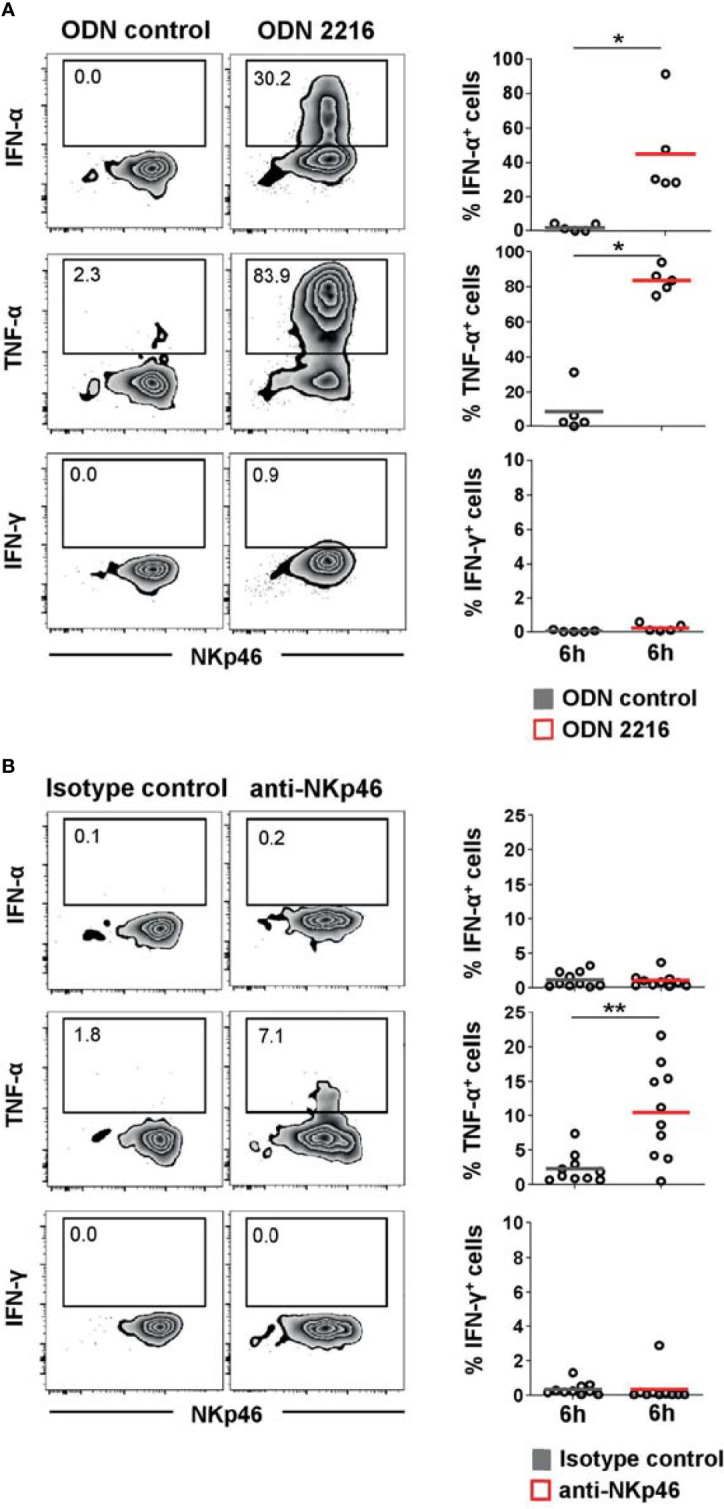
Cytokine induction in pDCs after stimulation with TLR9 agonist or NKp46 triggering. Intracellular staining of IFN-α, TNF-α, and IFN-γ was performed in PBMC of individual animals and is shown for CD4^high^CD172a^low^ pDCs. **(A)** PBMC were stimulated for 6 h either with the TLR9 agonist ODN 2216 (red) or incubated with non-stimulatory oligonucleotides (ODN control, gray). **(B)** Induction of cytokine production by receptor triggering was assessed in response to cross-linking of NKp46 after a 6-h incubation of PBMC with plate-bound mAbs (red). Plates coated with isotype-matched irrelevant antibodies served as control (gray). **(A, B)** Zebra plots on the left show cytokine production after stimulation for one representative animal; percentages of cytokine producing cells are indicated in the graphs. Frequencies of cytokine-producing cells within pDCs are shown for analyses of five **(A)** or ten **(B)** animals in the graphs on the right. Mean values are represented by colored bars. Significant differences between stimulated and non-stimulated cells are indicated (*p ≤ 0.05, **p ≤ 0.01).

As shown in previous studies, porcine pDCs are potent producers of IFN-α and TNF-α and can also produce IFN-γ in response to viruses and TLR agonists (7, 10). We could confirm a vast increase of IFN-α^+^ cells within porcine pDCs after TLR9 agonist stimulation (**Figure 3A**, upper row, ODN 2216: 45.2 ± 27.1%, red horizontal bar) compared to unstimulated cells (1.9 ± 2.1%, gray). Likewise, ODN 2216 induced TNF-α production in porcine pDCs (ODN 2216: 83.9 ± 7.1%, control: 8.4 ± 12.8%, **Figure 3A**, middle row), whereas no IFN-γ production could be observed (**Figure 3A**, bottom row). To investigate a possible role of the activation of NKp46 in cytokine production by porcine pDCs, cells were cultured in 96-well plates coated with anti-NKp46 mAbs (red) and compared to cells incubated in plates coated with isotype-matched control antibodies (gray). A small population of TNF-α^+^ pDCs could be induced after NKp46 receptor triggering in most animals analyzed. As this increase was only moderate in some animals, we analyzed a larger cohort of animals for this experimental setup to confirm statistical significance (**Figure 3B**, middle row, anti-NKp46: 10.5 ± 6.8%, control: 2.3 ± 2.1%). No obvious induction of IFN-α as well as IFN-γ was achieved by NKp46 receptor triggering (**Figure 3B**, upper and bottom row, respectively). To investigate a potential co-stimulatory effect of NKp46 triggering on cytokine production, NKp46 cross-linking was combined with ODN stimulation. Cells were cultivated in either anti-NKp46 or isotype-control-coated plates and additionally stimulated with either ODN 2216 or ODN control. In accordance with the data obtained when only NKp46 was triggered (**Figure 3B**), a small subset of TNF-α^+^ pDCs was found in NKp46-coated microcultures combined with the ODN control (**Supplementary Figure 3A**, anti-NKp46: 6.1 ± 3.4%, control: 2.1 ± 1.9%). No co-stimulatory effect of NKp46 triggering on IFN-α or TNF-α production was found in cultures that were stimulated additionally with ODN 2216 (**Supplementary Figure 3B**).

### Triggering of NKp46 Leads to Activation of Porcine pDCs

**Figure 4:**
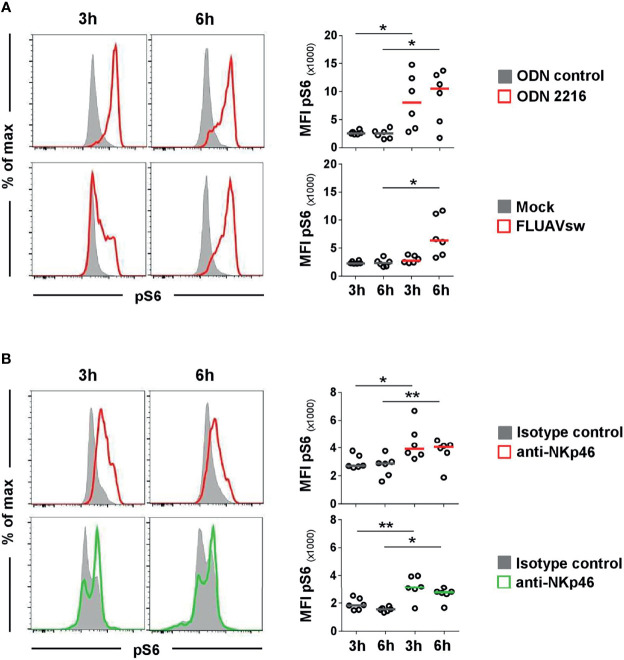
Flow cytometric analyses of phosphorylated ribosomal protein S6 (pS6) after stimulation of pDCs and NK cells. Induction of pS6 in pDC and NK-cell populations was assessed by analyzing the median fluorescence intensity after *in vitro* stimulation of PBMC. Phosphorylated S6 was detected intracellularly after fixation and permeabilization of cells by multicolor FCM. **(A)** For detection of pS6 in pDCs, PBMC were incubated with the TLR9 agonist ODN 2216 (red, upper row) or with a FLUAVsw H1N2 isolate (red, bottom row) for 3 and 6 h. Cells incubated with non-stimulatory oligonucleotides (ODN control, gray, upper row) or mock (gray, bottom row) served as corresponding controls. **(B)** Activation by NKp46 triggering of pDCs (red, upper row) and NK cells (green, bottom row) was assessed after a 3- and 6-h incubation of PBMC with plate-bound NKp46 mAbs. Plates coated with isotype-matched irrelevant antibodies served as controls (gray). PBMC for NK-cell analyses were additionally pre-activated with rpIL-2 and rpIL-15 for 24h. **(A, B)** Histograms show results of pS6 induction for one representative animal. Plasmacytoid DCs and NK cells were identified by phenotyping as outlined in **Figure 2A**. MFIs of pS6 within pDCs and NK cells are shown for analyses of six animals in the graphs on the right. Median values are represented by colored bars. Significant differences between stimulated and non-stimulated cells were calculated within the same incubation period and are indicated (*p ≤ 0.05, **p ≤ 0.01).

To investigate if NKp46 in pDCs is connected to functional signaling pathways, activation of cells was measured by using phosphorylation-specific monoclonal antibodies. We chose to monitor phosphorylation of the ribosomal protein S6, as it is a near-terminal response element in Ras/ERK and mTOR signaling and was observed to be a general activation marker for a variety of cell types and stimuli (34–36), including triggering of activating receptors of the NCR family (37). To establish phospho-specific staining, PBMC were stimulated with either ODN 2216 or influenza virus, the latter also shown to be a potent activator of porcine pDCs *in vitro* (38). CD4^high^CD172a^dim^ cells were analyzed for induction of phosphorylation of S6 (pS6) after 3 and 6 h *in vitro* stimulation (**Figure 4A**, red histograms). Cells incubated with ODN control or mock served as negative controls (**Figure 4A**, gray histograms). An increase of pS6 within pDCs after ODN 2216 stimulation was observed after a 3-h incubation compared to cells cultured in ODN control, indicated by a rise in the MFI of pS6 (ODN control: 2,563 ± 351, ODN 2216: 8,065 ± 4,908, **Figure 4A**, upper row, 3 h). Phosphorylation of S6 increased further after 6 h of cultivation in most of the animals (MFI ODN control: 2,498 ± 816, MFI ODN 2216: 10,500 ± 4,801, **Figure 4A**, upper row, 6 h). Only a minor induction of pS6 was detected in pDCs after stimulation with influenza virus (MFI: 2,754 ± 652) compared to the mock control (MFI: 2,299 ± 263) after a 3-h stimulation (**Figure 4A**, bottom row, 3 h). However, a more prominent induction of pS6 was observed after a 6-h incubation with virus (MFI: 6,509 ± 3,537) compared to mock control (MFI: 2,351 ± 680, **Figure 4A**, bottom row, 6 h). No obvious induction of pS6 was observed in NK cells after virus stimulation at both time-points (**Supplementary Figure 4**, green) compared to mock control (gray).

To test for NKp46 signaling activity, total PBMC were incubated in 96-well plates coated with anti-NKp46 mAbs for receptor triggering. Cells incubated in plates coated with isotype-matched control antibodies served as controls. To analyze activation of pDCs, CD4^high^CD172a^dim^ cells were analyzed for pS6 (**Figure 4B**, upper row) and compared to CD3^-^CD8α^+^ NK cells (**Figure 4B**, bottom row). A clear induction of pS6 could be observed in NK cells after 3 h of NKp46-triggering (MFI: 3,142 ± 857, green) compared to controls (MFI: 1,846 ± 421, gray). A similar 1.7-fold increase in pS6 expression could be observed after a 6-h NKp46-receptor triggering in NK cells (MFI NKp46: 2,770 ± 502, MFI control: 1,545 ± 156). Also in pDCs, an induction of pS6 was observed after NKp46-receptor triggering. A 1.5-fold increase was seen after a 3-h NKp46 stimulation (MFI NKp46: 4,380 ± 1,293, MFI control: 2,958 ± 516) and a 1.4-fold increase after a 6-h stimulation (MFI: NKp46: 3,738 ± 960, MFI control: 2,696 ± 765).

**Figure 5:**
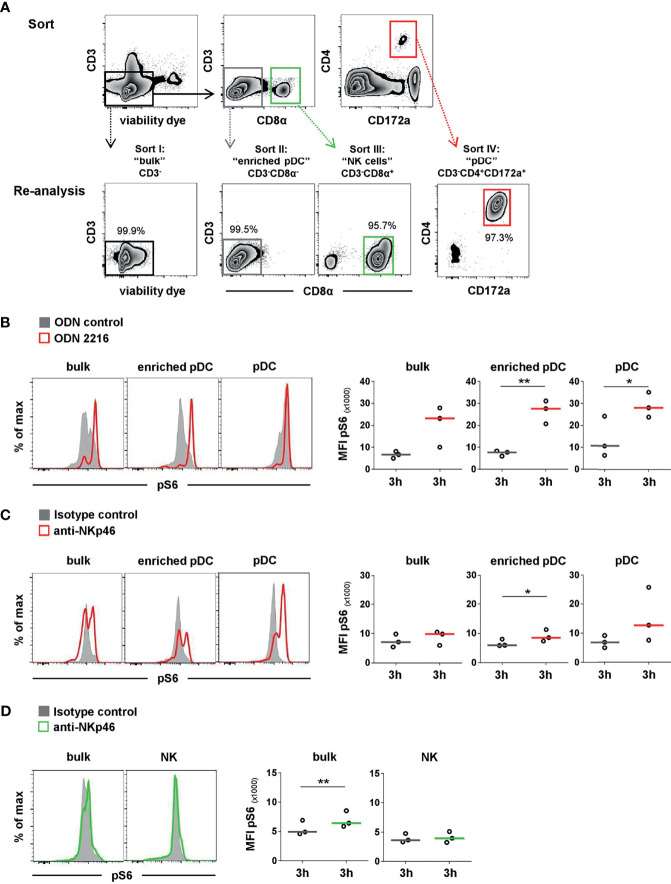
Activation of sorted pDC and NK-cell populations by NKp46 receptor triggering. **(A)** PBMC were depleted of T cells by MACS (CD3^-^, data not shown) and further sorted by FACS into four populations. I: non-T cells (CD3^-^), representing “bulk” culture; II: CD3^-^CD8α^-^, representing “enriched pDC”; III: CD3^-^CD8α^+^ “NK cells”; IV: CD3^-^CD4^+^CD172a^+^ “pDC”. Obtained purity of sorted populations was analyzed, and a representative reanalysis is shown in the zebra plots at the bottom. Induction of pS6 within gated pDC of NK populations (bulk, enriched) or pure pDC and NK-cell populations was assessed after 3 h *in vitro* stimulation. **(B)** Cells were incubated with the TLR9 agonist ODN 2216 (red) or with non-stimulatory oligonucleotides (ODN control, gray). Activation by NKp46 triggering of **(C)** pDCs (red) and **(D)** NK cells (green) was assessed after incubation with plate-bound NKp46 mAbs. Plates coated with isotype-matched irrelevant antibodies served as controls (gray). Cells for NK-cell analyses were additionally pre-activated with rpIL-2 and rpIL-15 for 24 h. **(B–D)** Histograms show results of pS6 induction for one representative animal. MFIs of pS6 within pDCs and NK cells are shown for analyses of three individual animals in the graphs on the right. Median values are represented by colored bars. Significant differences between stimulated and non-stimulated cells are indicated (*p ≤ 0.05, **p ≤ 0.01). **(B, C)** In “bulk” and “enriched pDC” cultures, pDC were gated according to a CD4^+^CD172a^+^ phenotype. **(D)** In “bulk” cultures, NK cells were gated according to a CD8α^+^ phenotype.

To confirm that the observed pS6 induction by pDCs was a direct outcome of NKp46 triggering and not due to a cross talk resulting from NK or NKp46^+^ T-cell activation in the PBMC culture, we performed further experiments on sorted cell populations (**Figure 5**). For this purpose, MACS CD3-depleted PBMC were FACS-sorted into CD3^-^CD4^+^CD172a^+^ pDC and CD3^-^CD8α^+^ NK-cell populations and compared to CD3^-^ bulk cultures, containing both populations (**Figure 5A**). Additionally, CD3^-^CD8α^-^ cells were investigated, being enriched for pDC but devoid of NK cells or NKp46^+^ T cells. To ensure functionality of pDCs after the sorting procedure, ODN stimulation assays were performed (**Figure 5B**). Additionally, FMO controls were applied to prove that observed increases in pS6 MFI values were not the effect of increased autofluorescence of stimulated cells compared to unstimulated controls (**Supplementary Figures 5A–C**). Three-hour stimulation assays on sorted cells with ODNs and plate-bound mAbs were performed, as previous experiments indicated optimal pS6 induction at this time-point (**Figure 4**). A clear induction of pS6 expression after ODN 2216 stimulation was observed in pDCs within the investigated sorted subsets (MFI bulk: 23,189 ± 9,269, MFI enriched pDC: 27,626 ± 5,313, MFI pDC: 28,170 ± 5,730, red) compared to ODN control (MFI bulk: 6,634 ± 1,564, MFI enriched pDC: 7,683 ± 1,224, MFI pDC: 10,744 ± 9,343, gray). After NKp46 triggering, pS6 was induced in pDCs in all three sorted conditions compared to controls, although only statistically significant in the enriched pDC group (**Figure 5C**). In the sorted bulk culture, on average a 1.4-fold increase was detected, similar to unsorted cells (**Figures 5C** and **4B**). Likewise, in the enriched pDC as well as pure pDC populations, an increase in the pS6 MFI was detected after receptor triggering (MFI enriched pDC: 8,520 ± 2,017, MFI pDC: 12,693 ± 6,444, red) compared to control (MFI enriched pDC: 5,936 ± 1,242, MFI pDC: 6,854 ± 2,100, gray; **Figure 5C** and **Supplementary Figure 5D**), thus resulting in a 1.4- and 1.9-fold increase, respectively. Induction of pS6 in NK cells was observed in the bulk culture after NKp46 triggering (MFI NKp46: 6,420 ± 1,396, green; MFI control: 4,914 ± 1,265, gray, 1.3-fold), and similar results were seen as in unsorted cells (**Figures 5D** and **4B**). Interestingly, this effect was not induced in the sorted pure NK population (MFI NKp46: 3,959 ± 935, green; MFI control: 3,642 ± 760, gray). Taken together, these results indicate a functional role of the NKp46 receptor in porcine pDCs.

## Discussion

In addition to the role of the activating receptor NKp46 on a subset of porcine NK cells (25, 26) and expression on a subset of non-conventional T cells with NK-associated functions (27), we show here that NKp46 is also expressed on another subset of innate immune cells in the pig. The high expression of NKp46 on the vast majority of porcine pDCs is unique compared to other species. The population of CD4^high^NKp46^+^ cells was clearly identified as porcine pDCs as they share a CD172a^dim^ phenotype, were assigned to large mononuclear cells due to their light-scatter properties, and were able to produce high amounts of IFN-α after TLR9 stimulation.

Although belonging to the myeloid subset, pDCs share some lymphoid features. Reizis recently highlighted in a review article the unique transcriptional program that associates pDCs in a “lymphoid-like” state and that pDCs show transcriptomic similarity to lymphoid progenitors (39). Up to date, no expression of NKp46 on CD4^+^ cells under steady-state conditions has been reported in the pig or other species, despite two reports on NKp46 expression on CD4^+^ lymphocytes in malignant cutaneous T-cell lymphoma (40, 41). So far, only the expression of the NCR family member NKp44 was reported on a minor fraction of pDCs in human tonsils *ex vivo* and in a fraction of human blood-derived pDCs after IL-3 stimulation. In contrast to NK cells, NKp44 receptor triggering has an inhibitory effect in human pDCs as decreased IFN-α production was observed in these cells (32, 33). Different from NKp46, NKp44 expression was not observed on porcine blood-derived pDCs *ex vivo*.

Shared features of DCs and NK cells were formerly described in mice for a subset of cells named interferon-producing killer dendritic cells (IKDC). These cells were phenotypically distinguishable from pDCs and conventional DCs, were able to kill NK-target cells, and produced IFN-γ (42, 43). In contrast, only a minor fraction of IKDCs was able to produce IFN-α. Cells with the described IKDC phenotype were also reported to express the activating receptor NKp46 (44). Nevertheless, subsequent studies argued for a redefinition of these cells and provided evidence that these IKDCs are highly activated NK cells or represent an intermediate NK-cell differentiation stage rather than belonging to the DC family (44, 45). For the porcine NKp46^+^ pDCs observed in our experiments, we can rule out that they represent a NK-cell subset or a similar DC-related IKDC population as no IFN-γ production could be observed after NKp46 stimulation that would be typical for these cell subsets. Furthermore, no perforin expression was observed in the NKp46^+^ pDCs *ex vivo*. It was reported that human pDCs are able to produce granzyme B, usually a hallmark of cytolytic T cells and NK cells. Granzyme B was induced *in vitro* by IL-3/IL-10 and seems to have an inhibitory role on T-cell proliferation (46, 47). Similar to our findings, these pDCs did not express perforin. It will be interesting to investigate a potential induction of granzyme B, or even perforin, by cytokines or even NKp46 triggering in porcine pDCs. This will be in the scope of a future study.

A topic of discussion is if the NKp46 molecule expressed by pDCs differs from the one expressed by NKp46^+^ lymphocytes in the pig. So far, alternative splicing variants for porcine NKp46 have not been reported, although three non-synonymous single-nucleotide polymorphisms and one deletion of three nucleotides in the coding sequence of the porcine *NCR1* gene were reported, which may affect the function of NKp46 (48). The functional role of NKp46 was already shown in regard to NKp46-induced IFN-γ production and degranulation by NK cells, whereas CD3^+^NKp46^+^ lymphocytes showed receptor-induced degranulation but no obvious cytokine production (26, 27). Functionality of NKp46 on porcine pDCs could be shown by triggering of the receptor by plate-bound NKp46-specific mAbs and induction of pS6, a molecule involved in protein synthesis at the ribosome (34) which is also activated downstream of the NKp46 signaling cascade (37). Similar results were observed for NK cells, at least in bulk cultures, similar to results described above (26, 27).

IL-15 is a well-known activator of the mTOR pathway, and stimulation with it leads to phosphorylation of pS6 in NK cells (49). We likewise observed basal pS6 induction in IL-15 primed NK cells already without NKp46 cross-linking (**Figure 4B**). Nevertheless, this pre-activation of NK cells is reported to improve NK-cell effector functions (50) and is also needed for receptor triggering in porcine NK cells, even in bulk cultures (26, 27). Assays for pDCs were performed without IL-15 priming in order to corroborate the observed pS6 induction as an effect of NKp46 triggering. Of note, pS6 induction was also observed in sorted/enriched pDCs after receptor triggering. Although only statistically significant in the enriched pDC samples, the induction was observed for all three animals to varying degrees also in bulk cultures and pure pDC cultures (**Supplementary Figure 5D**), thus demonstrating a functionality of NKp46 in this myeloid cell subset and ruling out that the observed pS6 induction is due to cross talk with NKp46^+^ NK cells or T cells. Surprisingly, in contrast to unsorted or bulk populations, the effect of NKp46 triggering in sorted NK cells was highly diminished. One possible explanation for this observation might be that porcine NK cells are more prone to a phenomenon, referred to as “sorter-induced cellular stress,” and the accompanied metabolic disturbances of cells (51, 52) or that for effective NKp46 triggering NK cells need co-stimulation in addition to cytokines. Taken together, this suggests that NKp46-mediated activation on distinct cell populations is dependent not only on cytokines but also on co-stimulatory receptors and cross talk to other cells. Interplays were also observed for human NK-cell activation by receptor triggering (53).

Although no induction of interferons was observed, a small subset of TNF-α-producing pDCs was found following NKp46 stimulation. Therefore, this activation pathway might contribute to TNF-α production in porcine pDCs. It is well known that *in vitro* stimulation of porcine pDCs with influenza virus leads to induction of cytokines, including TNF-α (10, 38, 54, 55). Plasmacytoid DCs recognize influenza RNA by their TLRs (56). NKp46 is reported to recognize hemagglutinins (HA) of influenza, parainfluenza, and Sendai virus, and ligation leads to activation of murine and human NK cells followed by lysis of influenza-infected cells (21, 57, 58). Likewise, porcine NKp46 can bind to influenza HA (24), indicating a role in sensing influenza infections. Nevertheless, the induction of pS6 in porcine pDCs following stimulation of cells with influenza virus observed in our experiments is more likely to be the effect of the TLR-mediated activation as no pS6 induction could be observed in NK cells in this setup using free virions. This missing activation of NK cells can be explained by the fact that dimerization seems to be essential for NKp46 activation (37, 59), as achieved by plate-bound mAbs. In contrast, the addition of free viral particles to cell cultures might be insufficient for receptor cross-linking of NKp46. Furthermore, it was shown that although engagement of influenza-infected target cells leads to killing in a NKp46-dependent manner, exposure to free virions, like in our setup, can lead to inhibition of NK-cell activity (60, 61). As NKp46^+^ CD3^-^ as well as CD3^+^ cells are enriched in the lungs of influenza-infected pigs (24, 27), a role of the porcine NKp46^+^ pDCs in the context of influenza-infected cells seems to be likely. Binding of NKp46 to HA might contribute to release of TNF-α in infected tissue as induction of a small population of TNF-α-producing pDCs could be observed after NKp46 cross-linking. To further elucidate if triggering of NKp46 leads to upregulation of other genes involved in pDC effector function or maybe maturation, RNA-Seq experiments of sorted pDC after stimulation will be beneficial. This approach was recently performed on porcine pDCs after stimulation with different TLR ligands, showing induction of chemokine receptors, co-stimulatory molecules, and genes involved in cytokine response (62). Therefore, this topic will be addressed in future studies.

In summary, our data show that nearly all pDCs in the pig express the activating receptor NKp46 and that NKp46 triggering *via* antibodies leads to phosphorylation of the ribosomal protein S6. In addition, a subset of porcine pDCs responded by TNF-α production to NKp46 triggering. Hence, our data suggest that NKp46 expression in porcine pDCs contributes to pathogen sensing in this important cell subset of the innate immune system.

## Data Availability Statement

The raw data supporting the conclusions of this article will be made available by the authors, without undue reservation.

## Ethics Statement

The animal study of 5-week old animals was reviewed and approved by the institutional ethics committee and the national authority according to § 26 of Law for Animal experiments, Tierversuchsgesetz 2012 – TVG 2012 (reference number: bmwf GZ68.205/0005-II/3b/2014). Blood from slaughterhouse animals was taken after electric high-voltage anesthesia followed by exsanguination, a procedure that is in accordance with the Austrian Animal Welfare Slaughter Regulation.

## Author Contributions

KM, AS, and WG were responsible for the conception and design of the study. KM, MS, and MR performed the experiments. KM analyzed the data and wrote the manuscript. WG and AS interpreted the data and supervised the study. All authors contributed to the article and approved the submitted version.

## Funding

KM and MR were financially supported by the Christian Doppler Research Association, Vienna, Austria, and Boehringer Ingelheim Vetmedica GmbH within the “Christian Doppler Laboratory for Optimized Prediction of Vaccination Success in Pigs.” The financial support by the Austrian Federal Ministry for Digital and Economic Affairs, the National Foundation for Research, Technology and Development, and the Christian Doppler Research Association is gratefully acknowledged. The funder was not involved in the study design, collection, analysis, interpretation of data, the writing of this article, or the decision to submit it for publication.

## Conflict of Interest

The authors declare that the research was conducted in the absence of any commercial or financial relationships that could be construed as a potential conflict of interest.

## Publisher’s Note

All claims expressed in this article are solely those of the authors and do not necessarily represent those of their affiliated organizations, or those of the publisher, the editors and the reviewers. Any product that may be evaluated in this article, or claim that may be made by its manufacturer, is not guaranteed or endorsed by the publisher.
