# Supplementary material for: Porcine Plasmacytoid Dendritic Cells Are Unique in Their Expression of a Functional NKp46 Receptor
Source: Front Immunol. 2022 Mar 11;13:822258. doi: 10.3389/fimmu.2022.822258 (PMC8970115; doi:10.3389/fimmu.2022.822258)
Supplement: Supplementary file 1 [file DataSheet_1.pdf]

Supplementary Material

Porcine plasmacytoid dendritic cells are unique in their expression of a functional NKp46 receptor

Running title:
NKp46^{+} porcine plasmacytoid dendritic cells

Authors:
Kerstin H. Mair[1,2*], Maria Stadler[1], Mahsa Adib Razavi[2], Armin Saalmüller[1] and Wilhelm Gerner[1,2,†]

Affiliations:
[1]Institute of Immunology, Department of Pathobiology, University of Veterinary Medicine Vienna, Vienna, Austria
[2]CD Laboratory for Optimized Prediction of Vaccination Success in Pigs, Institute of Immunology, Department of Pathobiology, University of Veterinary Medicine Vienna, Vienna, Austria
[†]present address: The Pirbright Institute, Woking, United Kingdom

***Corresponding author:**
Kerstin H. Mair
Institute of Immunology, Department of Pathobiology
University of Veterinary Medicine Vienna
Veterinaerplatz 1
1210 Vienna
Austria
Kerstin.Mair@vetmeduni.ac.at

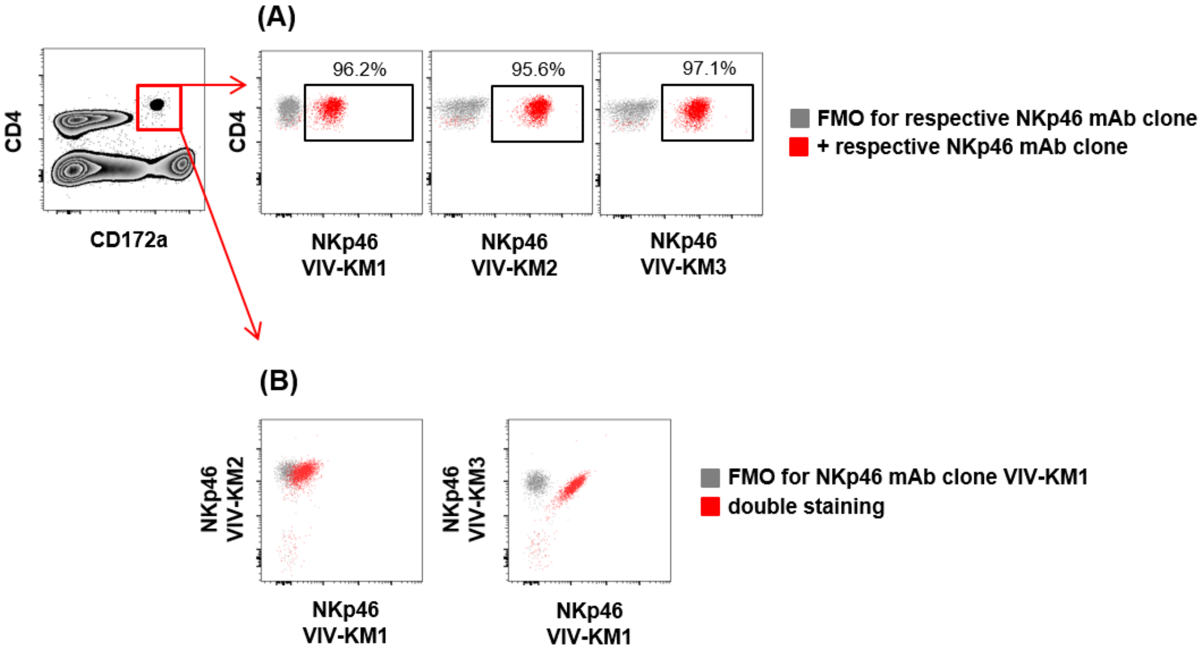

Supplementary Figure 1: Flow cytometric staining of porcine pDC with different anti-NKp46 mAb clones. $CD4^{high}CD172a^{low}$ pDCs (red gate) were analyzed for NKp46 expression in flow cytometry using three different NKp46-specific mAb clones: VIV-KM1, VIV-KM2, and VIV-KM3 (all mouse IgG1, in-house preparation). **(A)** The different mAb clones were applied in separate samples. Gray color indicates FMO controls for NKp46 of the different samples, red color represents samples where anti-NKp46 mAbs were added. Frequencies of $NKp46^{+}$ cells within gated pDCs are indicated. **(B)** Co-staining using mAb clone VIV-KM1 in combination with either VIV-KM2 or VIV-KM3. Gray color indicates samples, where no mAb clone VIV-KM1 was applied, red color represents samples where both mAb clones were added. FCM staining was performed as described in Material and Methods (Flow cytometry assays and antibodies used in the study). The same antibodies and reagents were used as listed in Table 2 (*ex vivo* phenotyping, pDC panel I). For the double staining, mAb clone VIV-KM1 was used directly conjugated to Alexa647. The partial blocking in the co-staining with VIV-KM1 and VIV-KM2 was discussed earlier as being most likely due to closely adjacent binding sites on the receptor (25). No such effect was observed in the co-staining of VIV-KM1 and VIV-KM3. Data is representative for experiments performed with three individual animals.

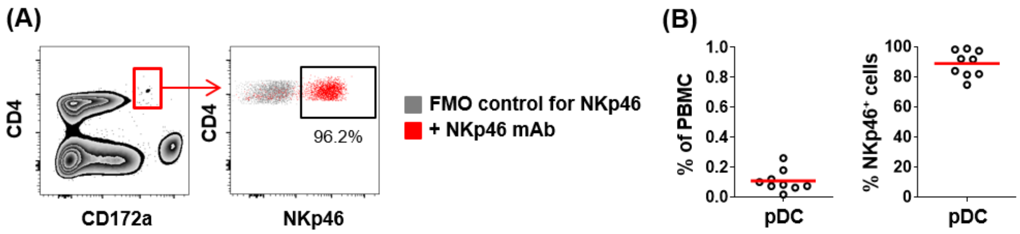

Supplementary Figure 2: (A) PBMC of 5-week old piglets were analyzed in flow cytometry. Cells were gated on $CD4^{high}CD172a^{low}$ pDCs (red gate) and further analyzed for their NKp46 expression. Data is shown for one representative animal, frequency of $NKp46^{+}$ cells is indicated. Gray color indicates FMO control for NKp46, red color represents samples where anti-NKp46 mAbs were added. **(B)** Frequencies of $CD4^{high}CD172a^{low}$ pDCs within total PBMC of all nine piglets analyzed are shown in the graph on the left. Frequencies of $NKp46^{+}$ cells within pDCs are shown in the graph on the right. Means are represented by colored bars.

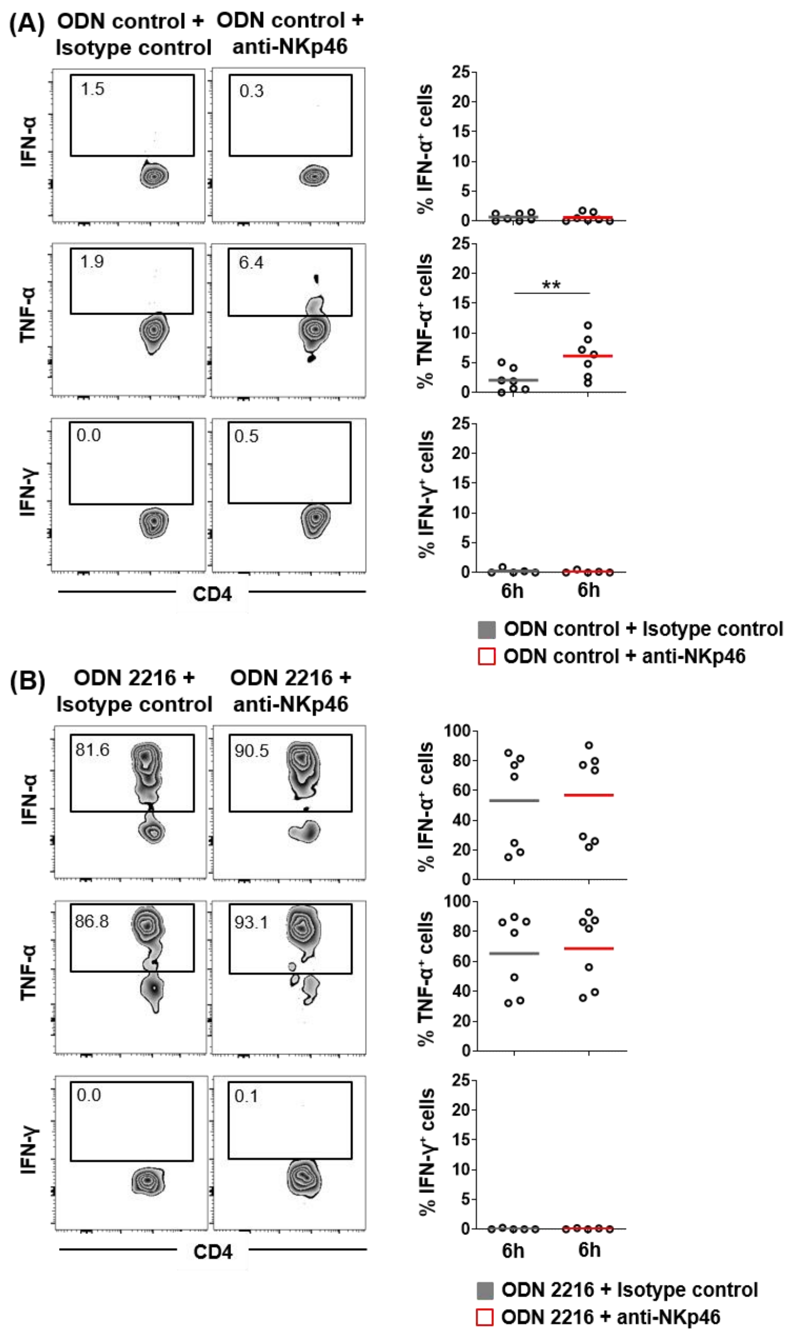

Supplementary Figure 3: Cytokine induction in pDCs after stimulation with TLR9 agonist in combination with NKp46 triggering. Intracellular staining of IFN-α, TNF-α and IFN-γ was performed in PBMC of individual animals and is shown for $CD4^{high}CD172a^{low}$ pDCs. PBMC were stimulated for six hours in the presence of ODN control **(A)** or the TLR agonist ODN 2216 **(B)** with either plate-bound anti-NKp46 mAbs (red) or isotype-matched irrelevant antibodies as control (gray). Zebra-plots on the left show cytokine production after stimulation for one representative animal, percentages of cytokine producing cells are indicated in the graphs. Frequencies of cytokine producing cells within pDCs are shown for analyses of five (IFN-γ) or seven (IFN-α, TNF-α) animals in the graphs on the right. Mean values are represented by colored bars. Significant differences between mAb stimulated and non-stimulated cells are indicated (** = $p \leq 0.01$).

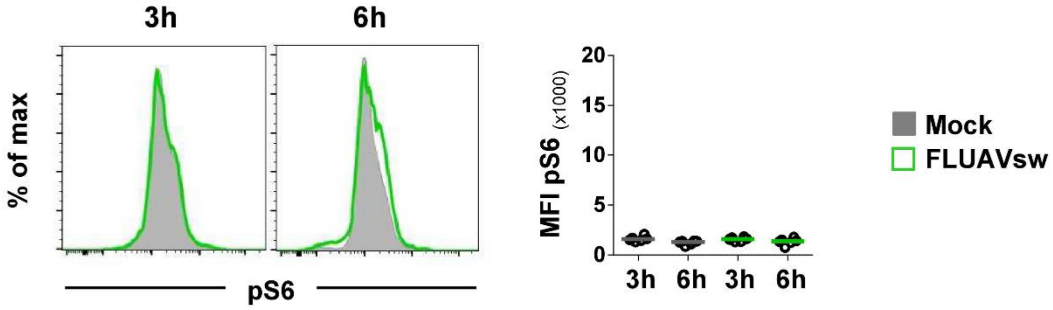

Supplementary Figure 4: Flow cytometric analyses of phosphorylated ribosomal protein S6 (pS6) after stimulation of NK cells with influenza virus. Induction of pS6 within $CD3^{-}CD8\alpha^{+}$ NK cells (green) was assessed after *in vitro* stimulation of PBMC with a FLUAVsw H1N2 isolate for three and six hours. Mock incubated cells served as corresponding controls (gray). Phosphorylated S6 was detected intracellularly after fixation and permeabilization of cells by multi-color FCM and the median fluorescence intensity was analyzed. Histograms show results of pS6 induction for one representative animal, MFIs of pS6 within NK cells are shown for analyses of six individual animals in the graph on the right. Median values are represented by colored bars.

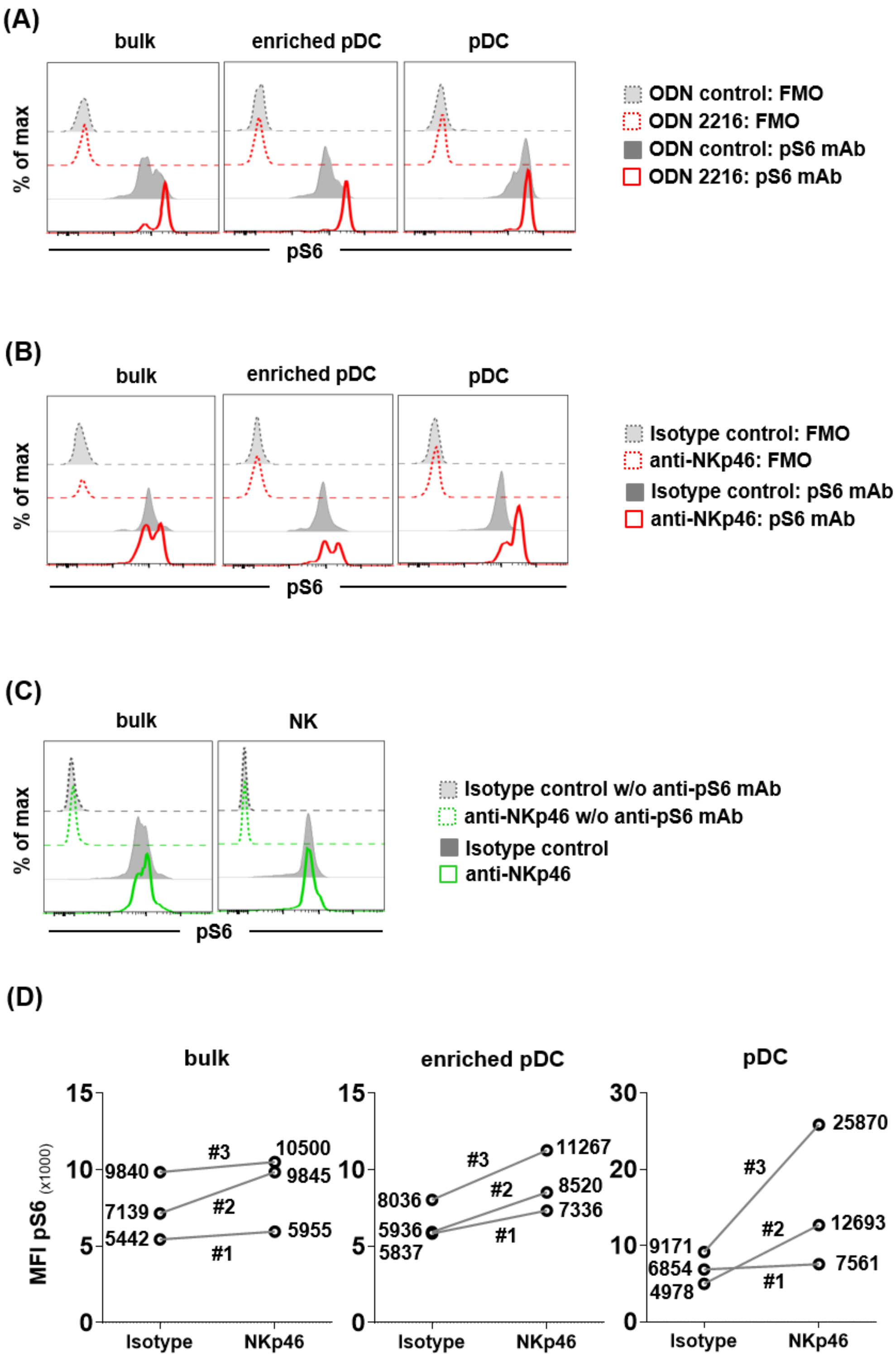

Supplementary Figure 5: FMO controls and MFI analysis for pS6 FCM staining. (A-C) Histogram overlays show the data of one representative animal from the sorting experiments as in Figure 5. FACS sorted cells were stimulated with **(A)** the TLR-9 agonist ODN 2216 (red) or non-stimulatory oligonucleotides (ODN control, gray), or **(B+C)** plate-bound NKp46 mAbs (red/ green) or isotype-matched irrelevant antibodies as controls (gray). Induction of pS6 was assessed after 3 hours

in vitro stimulation by multi-color FCM. Histograms with dashed lines represent the corresponding FMO controls to each sample. **(A+B)** In “bulk” and “enriched pDC” cultures pDC were gated according to a $CD4^{+}CD172a^{+}$ phenotype. **(C)** In “bulk” cultures, NK cells were gated according to a $CD8\alpha^{+}$ phenotype. **(D)** MFIs of pS6 within pDCs in the three sorted populations corresponding to the data shown in Figure 5C. Connecting lines indicate results obtained with cells from the same animal under the conditions tested.
